# Caregiver perspectives on barriers and facilitators to primary care for autistic adults: A qualitative study

**DOI:** 10.3389/fmed.2022.1022026

**Published:** 2022-11-10

**Authors:** Leah I. Stein Duker, Elizabeth Goodman, Amber Pomponio Davidson, Laura Mosqueda

**Affiliations:** ^1^Mrs. T.H. Chan Division of Occupational Science and Occupational Therapy, Ostrow School of Dentistry, University of Southern California, Los Angeles, CA, United States; ^2^Department of Health and Rehabilitation Sciences, College of Public Health, Temple University, Philadelphia, PA, United States; ^3^Family Medicine, Keck School of Medicine of the University of Southern California, Los Angeles, CA, United States

**Keywords:** environment, primary healthcare, autistic adults, primary care, autism

## Abstract

**Background:**

Primary care is associated with greater access to healthcare services and improved health outcomes. However, autistic adults report challenges accessing and utilizing primary care, in addition to unmet healthcare needs. The need to minimize existing barriers and identify strategies to facilitate successful healthcare encounters is increasingly important as autistic adults represent a growing segment of society. Minimal research has examined primary healthcare encounters for this population.

**Methods:**

As part of a larger convergent parallel design mixed-methods study that recruited autistic adults, caregivers of autistic adults, and primary care providers treating autistic adults, interviews were conducted with 31 caregivers of autistic adults. Caregivers were predominantly female (94%), and the autistic adult they cared for were primarily male (87%), with a mean age of 24 years. Thematic analysis was employed to elucidate the barriers to care, suggestions to mitigate challenges, and/or successful strategies implemented during care encounters for autistic adults, as reported by their caregivers.

**Results:**

Reported here are the results only from the caregiver interviews, in which seven themes emerged: (1) finding a primary care provider; (2) patient-provider communication; (3) anxiety due to unpredictability, an overstimulating sensory environment, and waiting time; (4) participation of consumers in the healthcare process; (5) stigma and assumptions about autism; (6) caregiver experiences; and (7) the impact of culture and ethnicity on care.

**Conclusion:**

Findings from this study have the potential to inform the development of, or improve existing, client-centered interventions to improve primary healthcare services for autistic adults.

## Introduction

Autism Spectrum Disorder (ASD) is a neurodevelopmental condition characterized by challenges with social communication and interaction as well as behavioral skills that affect daily functioning (1). The most recent report estimates that 1 in 44 children have been diagnosed with ASD (2).

Autistic adults^1^^,^^2^ experience elevated rates of medical and psychiatric conditions compared to the general population (3), are at increased risk for chronic disease (4), and are 3.4 times more likely than their non-autistic counterparts to be diagnosed with obesity and diabetes mellitus (5). As primary healthcare is correlated with increased access to healthcare services and improved health outcomes (6–8), it is particularly vital for this population to receive high-quality primary healthcare.

However, primary care experiences for autistic adults are often laden with challenges for the patient, their caregivers, and primary care providers (PCPs). Barriers to service utilization and high-quality, patient-centered care in the primary care setting for this population have been reported to include: (a) communication challenges; (b) a clinical physical environment that can be overstimulating to those with sensory sensitivities; (c) a lack of clarity on the part of health care practitioners about the proper channels for decision-making, including the roles of the patient and caregiver; (d) the influence of stigma pertaining to autism and incorrect assumptions about the patient's level of functioning; (e) lack of provider education concerning autism; and (f) administrative hurdles, such as financial disincentives for implementing needed accommodations, inadequate time for appointments, and limited reimbursement rates (9–17).

Considering these difficulties, it is not surprising that both autistic adults and caregivers of autistic individuals report heightened stress levels throughout the primary care process and diminished satisfaction with healthcare providers (10, 11, 18–22). In addition, some PCPs report feelings of inadequacy when providing care to autistic adults and desire autism-specific education in order to improve competency (9, 13, 14, 23–25).

Although a few interventions have shown preliminary efficacy to improve primary care health experiences for the unique needs of autistic adults [e.g., the Autism Healthcare Accommodations Tool (AHAT) (26); a phone-based pre-visit assessment to create individualized plans (15); the Extension for Community Healthcare Outcomes (ECHO) Autism Transition program (27)], there has been difficulty with widespread dissemination and implementation, likely due to barriers at the healthcare systems level (28). Additionally, PCPs, autistic adults, and caregivers of autistic adults report employing strategies only sparingly during primary care encounters, despite stating that they are helpful when used (29).

As autistic adults represent a growing segment of society (30), it is increasingly important to minimize existing barriers to successful primary healthcare encounters. In order to inform future service delivery techniques and improve healthcare services, it is essential to identify facilitators to care and explore the perceptions of service users (17). Therefore, the purpose of this study was to elucidate the barriers and facilitators to primary care health encounters, as reported by caregivers of autistic adults, to identify successful client-centered strategies to facilitate care as well as inform the development of new or improve existing interventions.

## Materials and methods

### Study design

This convergent parallel design mixed-methods study recruited autistic adults, caregivers of autistic adults, and primary care providers reporting to treat autistic adults. In the larger study, separate interviews were conducted with caregivers (as reported here), autistic adults, and primary care providers. Due to the richness of the data, results from the other respondent groups will be described in other publications. Reported here is a portion of the larger study, a qualitative inquiry which utilized interviews of caregivers of autistic adults to describe the barriers and facilitators they and their autistic adult child experienced during primary care health encounters. This study was approved by the institutional review board of the University of Southern California Health Sciences (HS-17-00477); informed consent was obtained from all participants.

### Participants

The majority of caregivers in this study were recruited when their autistic child could not participate in an interview verbally, written, or utilizing augmentative and alternative communication (AAC) strategies, in order to obtain information about primary care health experiences of autistic adults–albeit from the perspective of the caregiver. Four autistic adults who completed interviews requested their caregiver also be interviewed, so there are a small number of dyads present in the overall data. *Note:* the participant inclusion criteria reported below applies only to the caregiver group, as the other respondent groups are not included in this analysis.

To participate, caregivers had to support an adult 18 years or older with a diagnosis of ASD [confirmed by documentation from a medical professional or a score >65 on the Ritvo Autism Asperger Diagnostic Scale-Revised (31)], have previously accompanied the autistic adult to at least one primary care medical visit, and speak English or Spanish. Caregivers in Southern California and Philadelphia who responded to brochures/flyers, presentations at local sites (e.g., support groups, therapy locations, supported employment locations), and/or social media postings were enrolled in the study using a consecutive sampling technique. All participants enrolled completed the study.

### Data collection

Interviews followed a semi-structured guide with open-ended, narrative questions designed to elicit rich stories of primary care experiences, barriers and facilitators to care, and potential strategies for success/best practices. Questions were crafted by the authors based on the current literature available, gaps in the literature, and expert experience working with autistic individuals. These questions were then reviewed and edited by one expert in adult primary care, one expert in qualitative methodology, and two autistic adult stakeholders. Questions were then pilot-tested by five caregivers; revisions were made secondary to all reviewers' feedback. The final semi-structured interview guide included nine questions, including prompts about finding a PCP, frequency and reasons for PC visits, communication strategies during visits, barriers to primary care encounters, strategies utilized to make visits successful, and how primary care could be improved for autistic adults. Although the guide was used to direct the interview, additional probes were employed based on participants' verbal and nonverbal responses to questions. A copy of the script can be requested from the first author.

Interviews were conducted by three MA-level team members, all of whom had didactic and *in vivo* interview training as well as detailed protocol- and interview guide-specific training as it pertained to the current study. One interviewer was bilingual (English/Spanish) to enable the inclusion of participants who preferred to be interviewed in Spanish. Two of the interviewers were occupational therapists with experience working with autistic individuals. Interviews took place in the participant's home or in a private area in a location convenient to the participant (e.g., library) to minimize participant burden. All interviews were one-on-one (researcher-to-participant), with the exception of one husband and wife dyad. Each participant was interviewed once.

Interviews were digitally recorded and field notes were taken during the interview. Recordings were professionally transcribed verbatim and lasted an average of 34 min each (±15 min). Interviews conducted in Spanish (*n* = 3) were likewise transcribed verbatim, but then translated into English by a professional translator, with the translation double-checked for accuracy by the bilingual study team member. Participants were provided with a $30 debit card to compensate them for their time.

### Data analysis

Thematic analysis following a grounded theory approach (32) and a constructivist research paradigm (33) was employed to describe barriers and facilitators to primary care health encounters for autistic adults and their caregivers. Two members of the research team independently read and coded three transcripts before meeting to create a provisional list of codes and sub-codes developed inductively from the data. After discussing potential codes, another three transcripts were independently read to search for additional codes and sub-codes prior to the formalization of the codebook. Using the agreed upon list of codes a minimum of two team members independently coded each interview using QSR International's NVivo qualitative data analysis software. Discrepancies in coding were resolved through discussion in collaboration with a third team member until a consensus was reached. Techniques to support the credibility and trustworthiness of the findings included: analytic triangulation (independent co-coding), moderator supervision, negative case analysis, consensus-driven thematic development, maintaining an audit trail, and fidelity checks for interview techniques (34). All 21 items of the Standards for Reporting Qualitative Research (SRQR) guidelines were addressed (35).

## Results

Participants included 31 caregivers of autistic adults (one mother and father dyad participated in a joint interview; this dyad was counted as one caregiver). Caregivers primarily self-identified as White (74%) and not Hispanic/Latino (61%), with the primary language spoken at home English or multiple languages including English (77%). Caregivers reported that the autistic adult they supported was most often white (74%), not Hispanic/Latino (65%), with the primary language spoken at home English or multiple languages including English (77%); autistic adults were most commonly reported by their caregivers to communicate using sentences (61%) (see Table 1).

**Table 1:** Caregiver-report descriptive characteristics of caregiver participants and the autistic adult they care for.

	**Caregiver**	**Autistic adult**
	**(*n* = 31)**	**(*n* = 31)**
	**Mean (SD)**	**Mean (SD)**
**Age**	n/a	24.3 (6.6)
**RAADS-R score**	n/a	124.4 (31.5)
	***N*** **(%)**	***N*** **(%)**
Sex*		
Male	3 (9.7)	27 (87.1)
Female	29 (93.5)	4 (12.9)
Race**		
White, Caucasian	23 (74.2)	23 (74.2)
Black, African American	5 (16.1)	7 (22.6)
Asian	2 (6.5)	2 (6.5)
American Indian or Alaska Native	2 (6.5)	2 (6.5)
Not reported	1 (3.2)	1 (3.2)
Not a family member of autistic adults	1 (3.2)	n/a
**Ethnicity**		
Not Hispanic, not Latino	19 (61.3)	20 (64.5)
Hispanic, Latino	11 (35.5)	11 (35.5)
Not a family member of autistic adult	1 (3.2)	n/a
**Primary language spoken in the home**		
English	22 (71.0)	22 (71.0)
Spanish	5 (16.1)	5 (16.1)
More than one language***	4 (12.9)	4 (12.9)
**Highest level of education**
High school/GED or less	9 (29.0)	28 (90.3)
College	7 (22.6)	3 (9.7)
Graduate degree or above	14 (45.2)	0 (0.0)
Not reported	1 (3.2)	0 (0.0)
**Communicates needs or wants by using _______**		
Sentences	n/a	19 (61.3)
Single words/phrases	n/a	10 (32.3)
Augmentative and alternative communication device	n/a	2 (6.5)

Counts may not sum to 100% due to missing data. RAADS-R = Ritvo Autism Asperger diagnostic Scale-Revised.

*One father and mother both participated in interview.

**Participants instructed to mark all that apply.

***More than **one** language: English and Spanish (n = 2), English and Italian (n = 1), English and Korean (n = 1).

Seven themes emerged from the interviews: (1) finding a PCP; (2) patient-provider communication; (3) anxiety due to unpredictability, an overstimulating sensory environment, and waiting; (4) consumer inclusion in the healthcare process; (5) stigma and assumptions about autism; (6) caregiver experiences; and (7) the impact of culture and ethnicity on care (see Table 2).

**Table 2:** Endorsement of reported themes per caregiver participant.

**ID**	**Finding a PCP**	**Communication**	**Anxiety**	**Consumer inclusion**	**Stigma**	**Caregiver experience**	Culture*
01	X	X				X	
02	X	X	X	X		X	
03	X	X	X	X	X	X	X
04	X	X	X	X		X	
05	X	X	X	X	X	X	
06	X	X	X	X	X	X	
07	X	X	X	X	X		
08	X	X	X	X	X	X	X
09	X	X	X	X		X	
10	X	X	X	X	X	X	X
11	X	X	X	X	X	X	X
12	X	X	X	X	X	X	
13	X	X		X	X	X	
14	X	X	X	X		X	
15	X	X	X	X	X	X	
16	X		X	X	X	X	X
17	X	X	X		X	X	X
18	X	X		X		X	
19	X	X		X	X	X	X
20	X	X	X			X	X
21	X	X		X	X	X	X
22	X	X		X		X	
23	X	X		X		X	
24	X	X		X	X	X	
25	X	X	X	X	X	X	
26	X	X	X	X	X	X	
27	X	X		X			
28	X	X	X	X	X	X	
29	X	X	X	X		X	
30	X	X	X	X			
31	X	X	X	X	X		
**Percentage**	**100%**	**97%**	**71%**	**90%**	**61%**	**87%**	**56%**
**Endorsed**

*Shaded boxes indicate participants who self-reported to be Hispanic/Latino and/or Black/African American.

### Finding a PCP for autistic adults

#### Barriers

Almost half of participating caregivers (*n* = 15) reported challenges finding a PCP for their autistic adult child, with experiences ranging from being “a bit bumpy” to “very difficult.” These caregivers overwhelmingly stated that they “wouldn't even know how to shop for [a PCP],” and that there was insufficient access to “resources…that [are] like, ‘Hey…if your adult child has ASD, you can go. These are really wonderful doctors who are going to embrace you.”' In addition, one caregiver reported struggling to find PCPs willing to accept their autistic adult child into their practice, explaining that:

“*It's calling an office cold …. and they don't know you, and all they see is a large, young adult who looks like he might do something unexpected…I can't tell you how upset it made me to have medical practitioners turn him away. I didn't even know you could do that. I honestly didn't think that was a thing. But I discovered that it is*.”

Multiple participants reported finding care at teaching hospitals or clinics, only to experience fragmented and inconsistent care due to frequent fluctuations of PCPs. Although caregivers did report desiring a PCP who was “youngish, so…they'd be around a while,” others explained that their experience with a teaching practice was “a bust” because they “can't have a new doctor every two [years] or, however long internship is. That'll never work.” Another caregiver with a similar experience explained it was particularly burdensome when “you lose the thread…and you have to start again with a different doctor.” As perceived by caregivers, this constant changing of PCPs also created stress for autistic adults because they “[don't] like to switch up that much” because they can “take a while to get comfortable with people.” Some caregivers found PCPs through an insurance-provided network; however, participants sometimes considered this a barrier because the physicians covered by their insurance “generally speaking, don't have experience with this group of adults.” Additionally, one caregiver explained that the practice covered by her insurance was small and therefore it was “very difficult because you are only assigned one doctor and that's just the only doctor that's going to see you and nobody else. And then when we disagree with that doctor, there's not another doctor that would see us.”

#### Facilitators

Several caregivers reported finding a PCP for their autistic adult child through recommendations from their former pediatrician, family members in the medical field, coworkers, or other parents (*n* = 7). Caregivers emphasized the importance of these connections and explained that “within the community of ASD, you reach out to other individuals who are on the same journey as you.” One caregiver noted that an added benefit of speaking to a PCP that came highly recommended was that it made her feel as if she had interviewed the doctor before meeting them. Multiple caregivers also reported choosing their own already-trusted PCP to provide care for their autistic adult child (*n* = 5) in lieu of searching for a new provider. Caregivers identified familiarity as a key determinant when choosing to use their own PCP or to remain with their child's pediatrician into adulthood. Caregivers described that selecting their own PCP was a byproduct of both “… me knowing them…and because I've been going there for a while… they are nice to [my child]”, as well as being fearful and “afraid of having [my child] go to someone that I wasn't really familiar with.”

A few caregivers reported that their pediatricians were willing to continue to treat their autistic child into adulthood (*n* = 2), with one mother expressing that she was “eternally grateful” her pediatrician did this because she could not “imagine what my search would look like to find a primary care physician for an adult with autism,” adding that “it would take a lot of leg work in order to find somebody that would be suitable.”

Lastly, a few caregivers explained that their insurance companies provided a directory of PCPs, which made the process of finding a provider less overwhelming because “there's just three to choose from.” One caregiver expressed her gratitude for her private insurance because “they help you do that transition” from pediatrician to adult PCP. However, as stated earlier, these short lists of approved providers simultaneously posed challenges for other families.

##### Caregiver suggestions

The idea of a “facilitated facility” for adults with developmental disabilities “that you knew going in that they understood that they would make the accommodations” was suggested by caregivers. One caregiver described how “you have to ask for everything, and you have to prepare everything. But still, sometimes if you prepare ahead of time, they don't have the people that will help you to do certain processes.” This type of specialty clinic would aim to ameliorate an issue that an overwhelming number of caregivers reported as a significant barrier to the receipt of primary care—finding PCPs and staff with autism-specific training, experience, and understanding. In addition, caregivers noted that a specialty clinic would provide an increased sense of community for both autistic adults and caregivers. In addition, caregivers suggested this kind of facility could also employ specialists such as behavioral therapists to provide emotional support and assist in behavior management during health encounters if needed.

### Patient-provider communication

#### Barriers

Challenges with expressive and/or receptive communication were reported by caregivers as a barrier to patient-provider interactions during primary care encounters. Many caregivers stated that autistic adults experienced challenges with expressive communication; therefore, PCPs often relied on a combination of caregiver and patient descriptions in order to understand the medical status of their autistic patient, making these health encounters a “team effort.” As described by caregivers, some autistic adults were “not able to tell them [the physician] what she needs,” while others chose to not communicate with providers due to a “…lack of interest and not wanting to. It's like, I don't care about this, so I'm not going to answer you.” While caregivers often reported that their autistic adult child was able to answer concrete questions (e.g., water intake, medication list), challenges arose when PCPs asked subjective questions about how their patient felt. Multiple caregivers explained that their autistic adult child “can't express to you how he feels; just that he feels *weird*” and that they struggled to formulate responses beyond “I don't feel good.”

Caregivers reported that their autistic adult child also experienced challenges describing and expressing their pain. Some described that the autistic adult might “copy somebody” like the PCP and thereby misreport symptoms; for example, “if [the doctor] says, is your foot hurt here? He goes, yeah, it hurts right here. Does this foot hurt here [someplace else]? Yes, it hurts right here,” making his answers mimic the questions asked by the PCP. Ultimately, caregivers reported that “the hardest part for everybody is just kind of what's going on with him, it's sort of unknowable to all of us.” Other caregivers believed their autistic adult child had a high pain tolerance because “when he gets hurt, he doesn't react the way I would…he doesn't say anything.” Due to this lack of reporting, caregivers commonly explained that they only became aware of their autistic adult child's injuries after noticing visible symptoms such as blood, which delayed the onset of treatment.

Interestingly, a few caregivers noted that they believed PCPs overlooked consideration of communication-related accommodations for autistic adults without apparent expressive language challenges. One caregiver reported that her son's strengths expressing himself caused PCPs to “assume he's understanding at a [high] level, so people talk above his level a lot because he's so expressive that they make the assumption, incorrectly, that he receives language as well as he expresses it.” Another caregiver explained that “the kids in the middle are also tending to get lost, because their communication skills are very much affected, but they pass closely enough that they're not getting that deep state help that they need.”

#### Facilitators

##### Caregiver strategies

Communication-related strategies were less frequently implemented by caregivers and/or autistic adults during primary care health encounters. A few caregivers described the use of technology to augment patient-provider communication. For example, one mother advised her son to take health-related pictures on his phone to show his PCP, while another translated the notes her son wrote in his phone in order to facilitate his communication with the PCP.

##### PCP strategies

Though utilized infrequently during care, caregivers recounted some communication-based strategies that PCPs used to promote successful primary care encounters. These strategies included: (1) adapting their communication style to their patients; (2) employing time modifications; and (3) consulting with caregivers.

Caregivers reported that it was particularly valuable when PCPs tailored their communication style to be compatible with the autistic patient's abilities; for example, speaking at a speed that allowed enough time for processing, and/or using visuals rather than “doctor language to where [we leave] and don't understand anything he said.” Caregivers noted it was important for PCPs to be intentional with their choice of language when providing medical explanations in order not to condescend to autistic patients. One caregiver said they appreciated when their PCP “explains things thoroughly to [my daughter]…I don't think he talks down to her, but by the same token, he is careful to use language that she understands.” Another parent agreed that it was valuable when the PCP did not infantilize her son but:

“*Whatever the problem is…he just treats him like a person… he - I guess respects him. You know, he knows his disability and everything, so he actually has a conversation with him. Not just making decisions or talking to me and ignoring [my son].”*

PCPs allotting sufficient time during appointments was reported by caregivers to facilitate patient-provider communication. While it was acknowledged that physicians are under pressure to see a considerable number of patients throughout the day, and that “time is money for doctors,” caregivers lauded PCPs that “give [my child] time to talk” by not rushing the appointment and providing extra time to ask questions. One caregiver praised a PCP that “…didn't seem to be in a hurry. And that's kind of the last thing you need. You really want your kid to feel comfortable and you want your kid to have all of his or her questions answered.” Highly touted providers also distinguished themselves by spending time during appointments to take an interest in their patient's personal lives, “just asking questions. No matter how relevant it is…That I appreciate, because that shows that the doctor cares. That there's compassion. It's not just something he [wants to bill], and that's it.” Conversely, one caregiver explained that appointments that were too lengthy could be detrimental, explaining that “you've just kind of got to find that right timing.”

Consultation with caregivers was described as essential for healthcare encounters, especially when autistic adults experienced communication difficulties that impacted their ability to answer PCP questions and/or articulate their current health concerns. For example, one caregiver appreciated that her PCP would call or email her after the exam if her son could not provide an answer to a question at that moment. This was an especially effective strategy because the caregiver could rephrase the question to their autistic adult child which might elicit a more accurate response.

### Anxiety

#### Barriers

Challenges with anxiety during primary healthcare appointments were described in four ways: (1) the inherent unpredictability of the medical encounter; (2) as a result of previous traumatic medical experiences; (3) an overstimulating sensory environment; and (4) long wait times.

According to caregivers, the *unpredictable nature of primary healthcare encounters* was anxiety-inducing for autistic adults. Anxiety was heightened when autistic adults were unfamiliar with the physical office space and/or the PCP treating them. One caregiver explained that her autistic adult child did not feel comfortable unless he “knows what to expect. To know who is going to be there [in the office] and what it's going to look like.” Caregivers sought PCPs their family could develop long-lasting relationships with, noting that their autistic adult child had a higher chance of a successful visit if there was “the consistency of the same office, the same people.”

Some caregivers attributed their autistic adult child's challenges attending primary care appointments to *previous traumatic medical experiences*. For example, one caregiver explained that drawing blood was problematic for her son “because he had a bad experience…from way back when he was young, he remembers and he really is jittery.” Another caregiver shared that her daughter was restrained for an EEG as a young child and as a result, “she's still scared to go, even pass by the hospital. So she has a lot of fear regarding that and anxiety when we have to go [to primary care appointments]…she's still traumatized.” Consequently, caregivers believed their autistic adult child was hesitant to inform them that they felt sick in order to avoid a visit to the doctor.

A *sensory environment* incompatible with the autistic adults' sensory preferences hindered successful primary care experiences. Many caregivers reported that their autistic adult child disliked being touched, which made the physical examination and associated procedures difficult. Additionally, fluorescent, bright, and/or “strob[ing]” lights, loud noises, and the “crunch paper” on the examination table were bothersome, making the waiting room and examination room challenging. One caregiver described the physical environment at her child's PCP's office as “…overwhelming... The sounds. [The] lighting.”

An overwhelming majority of caregivers reported that the extensive amount of *time spent in the waiting room or exam room* prior to being seen by their PCP was “…the worst part of the whole [primary care] experience” for their autistic adult child and themselves. Waiting was described as “excruciating”, with one caregiver expressing that “the greatest favor any practitioner could do for us is not keep us waiting.” Waiting was a challenge due to its unpredictable nature, the exhibition of challenging behaviors by the autistic adult, and caregiver irritability due to staff not “be[ing] honest with me” in relation to wait times. Although multiple caregivers expressed that waiting in the examination room was preferable to the waiting room, most caregivers agreed that 15 to 20 min was the maximum total amount of time that should be spent regardless of location. When that time frame was exceeded, some caregivers stated that their autistic adult child “will start getting anxious and, you know, [act] up a little bit.”

#### Facilitators

##### Caregiver strategies

Caregivers recounted a few strategies they implemented that they perceived to mitigate their autistic adult child's anxiety throughout primary healthcare experiences. These strategies included: (1) strategic scheduling; (2) preparation before and debriefing after the encounter; (3) cognitive-based techniques, (4) sensory-based strategies, and (5) use of technology.

Caregivers' use of *strategically scheduling visits* was reported to increase the chances of a successful primary healthcare encounter. When making this decision, caregivers considered the optimal time of day they felt their autistic adult child functioned best and when their child's daily routine would be least disrupted. Some caregivers who chose the first appointment of the day explained it was because their autistic adult child “does better in the mornings…He's kind of lethargic and tired by afternoon” or that the early morning is when their medication is most effective. Caregivers also commonly described that healthcare visits that disrupted routines were problematic. One mother explained that “… I try and pick a time when he's not in school. Because to pull him out of school would stress him out beyond belief. So, if I can, I schedule it on a schedule[d] vacation,” while others described that changing the routine for a medical appointment made their autistic adult child “fidgety” and contributed to challenges during the encounter. When engaging in strategic scheduling, caregivers also considered when they believed the wait would be shortest. Both first and last appointments of the day were reported to facilitate a quick encounter with minimal waiting “so [we] didn't have to sit around in the waiting room” because if “there's no waiting around he doesn't complain. And he doesn't stress out. So, if he can get right in, that's really, really helpful.”

Caregivers spoke to the importance of *preparation before and debriefing after* healthcare encounters. For example, caregivers reported using social stories to prepare their autistic adult child for difficult and stressful procedures, describing that “little by little, we just put [it into] a story.” Another caregiver reviewed a schedule prior to the visit with her autistic adult child, describing that “‘First, we're going to the office, we're going to see the doctor, then we're going to get [you] treated.' Basically, just listing out the steps of what's going to happen and what the outcome is going to be.” In addition, caregivers reported describing the impending healthcare encounter “for days before we go there” and “preplan[ing] her questions and discuss[ing] them beforehand [so] she doesn't freeze up [and can] get all of her issues out there on the table and be heard and get answers.”

Post-visit debriefings were an opportunity to allay fears and enhance understanding of medical encounters. For example, one caregiver explained that when her son “said he's a little scared, I tell him ‘Don't worry about it, we can talk about this one later in the house,' ” while another described that “when she comes back, she processes with me and that's a really important thing, for her to have someone to process with.” Overall, this strategy was believed by caregivers to “ease the stress” of the primary care experience for autistic adults by fostering a deeper understanding of the medical process.

Caregivers recalled using *cognitive-based strategies* in efforts to reduce their autistic adult child's anxiety during the healthcare encounter. This looked like “talking it through, slowing it down. Stopping, letting him be in control”; “[redirecting] his emotions, like, wait, count to 10”; and “[talking] to him. Like, [trying] to engage him in a conversation that he likes.” One caregiver explained:

“*I just try to keep him calm. I acknowledge his frustration. I let him do what he needs to do… I never chastise him when he is loud for being loud, because he does it for a reason. But I do let him know and remind him that there are places where the voice is inappropriate. So I just am always reminding him, this is a place where you can't use that voice.”*

Targeted *sensory-based strategies* to minimize noxious auditory and visual sensory stimuli were utilized during healthcare encounters, as reported by caregivers. These included removing noisy examination table paper, using headphones for noise reduction, listening to music, and/or asking staff to dim the lights. Sensory-related tools were also reported to be used for self-regulation. For example, one caregiver described that “we made sure that those things [sensory tools and fidgets] came with us that he could use to self-regulate. To be allowed to bring in some of his, you know, key coping mechanisms” in order to minimize anxiety was beneficial.

*Technology* was also strategically used by autistic adults to cope with long wait times. Caregivers stressed the importance of coming to appointments equipped with electronic devices; smartphones and iPads were reported to mitigate anxiety and make extensive waits more tolerable by giving autistic adults something to “occupy” and “distract” themselves with. Many caregivers also noted that without these types of devices, meltdowns could ensue.

##### PCP strategies

Caregivers recalled four strategies that although rarely implemented by PCPs, they believed helped reduce their autistic adult child's anxiety, including: (1) affording autonomy to the autistic adult during the examination, (2) narrating the examination, (3) use of nontraditional examination locations and/or equipment, and (4) reducing wait times.

*Affording autonomy* was perceived by caregivers to provide autistic adults with a sense of control, promoting comfort and easing anxiety during the examination. One caregiver explained:

“*I think the care providers that are really in tune give him options, so it's like, “Do you want to sit up on the table, or would you rather stay there in the chair?”…or, “Would you like to hold this before I stick it on you?”… So giving him a sense of a little bit of control over a situation where he may not like what has to happen, but at least within it, there's a choice.”*

Caregivers also noted their gratitude for these techniques, explicating that “I really appreciate those providers that are…willing to make some accommodations or adaptations and think about, ‘Wherever I can, I'm going to give him a choice.”' This caregiver added that when PCPs initiated strategies such as providing choices during the exam—rather than relying on her (the caregiver) to do so—it transformed an otherwise “exhausting” experience into an “easy process.”

*Providing narration of the exam* was also helpful to reduce anxiety. Specifically, caregivers explained that it was important for PCPs to use clear and concise narration during the physical examination, such as explicitly explaining the procedure they will perform, and describing how and why they will use the medical instruments in “language that [my child] understands.”

*Use of a nontraditional location and/or equipment* was also endorsed by caregivers. One caregiver commended a PCP who performed examinations in their office on a sofa rather than on a traditional examination table and also did not wear “doctor clothes.” Both strategies were reported to make the visit less stressful for their autistic adult child who had a previous traumatic medical experience. One caregiver described that her daughter's tactile sensitivity made it impossible to get an accurate reading of her heart rate and blood pressure. Their PCP accommodated these sensory preferences by using a FitBit to record these values instead.

PCP accommodations that focused on *prioritizing short wait times* for autistic patients were reported to be important and successful, with multiple caregivers commending PCPs for making it “easy for all of us” by seeing patients immediately upon arrival to purposefully minimize wait time. However, several caregivers expressed disappointment that other waiting-related accommodations such as strategic scheduling for the first or last appointment of the day were not offered.

##### Caregiver suggestions

Caregivers stated that their ideal primary care office would feel “warm” and “homey” to reduce stress and anxiety about the process. Caregivers emphasized the importance of a welcoming and kind office staff, including the receptionists, nurses, and PCPs that “make you feel that you are a part of their family, the clinic's family.” One caregiver reported that this would be conducive to a successful visit because “if somebody makes me happy, they're going to make [my son] happy because he feeds off that.” This ideal primary care office would offer a separate “quiet room” to mitigate auditory overstimulation, provide distractions such as a television or coloring books, and supply calming visual and tactile sensory stimuli such as a fish tank or fidget toys. One caregiver suggested that it would be helpful if the waiting room provided:

“*…a sensory box where you can explore…rather than the parent having to bring the known items that are going to help keep that child regulated…and you can have a range that provide for an additional stimulation or the auditory or the tactile, proprioceptive, you know, having chairs that are actually balls rather than, you know, hard chairs, or, you know, squeeze vests or, you know, those heavy–Blankets that have, you know, the smell of lavender or just sensory-input items or sensory-calming items.”*

### Inclusion of consumers in the health care process

#### Barriers

##### Barriers for autistic adults

Caregivers often reflected on their desire for their autistic adult child to gain independence and play a greater role in care coordination, healthcare management, and medical decision-making. Several caregivers admitted dwelling on how they were “not going to be here forever” and as a result were “…trying to make [my child] be as independent as I possibly can” so their autistic adult child could eventually advocate for themselves.

Caregivers recognized that if coordinating care, managing healthcare, and making medical decisions were complex processes for them to navigate, it would also pose a challenge for their autistic adult child. Many caregivers anticipated this to be an exceptional challenge because they typically took responsibility for navigating this system; therefore, their autistic adult child was never tasked with managing their own health care and had not been taught skills such as filling out paperwork and making appointments. Some caregivers added that they also wished there was staff to guide their child in filling out their own medical history so they can eventually “take a back seat.” One caregiver explicated that autistic adults were often not equipped to manage their care independently because:

“*If they haven't been advocating for themselves ‘till they're 18, now, all of a sudden, on their 18th birthday, they're just not going to get all this wisdom and this knowledge and be able to advocate and ask the questions for - they're not going to get the knowledge that this mom has had or this dad has had for the past 18 years to be able to go in that room and be able to talk to them [PCPs and/or medical staff] themselves.”*

When reflecting on the inclusion of autistic adults in medical decision-making during primary care encounters, the majority of caregivers stated that their autistic adult child was, in some way, involved in the process. However, the level of involvement varied among respondents, based on the autistic adult's skills and the significance of the decision, with caregivers often describing that “…he is part of the team, …[but] we do most of it.” Other caregivers reported that their autistic adult child was never included in decision-making because they “can't really make an informed-consent decision” or because of the urgency and gravity of the medical situation. One caregiver aptly described the process of inclusion in medical decision-making as a “dance,” illuminating the challenges inherent to the inclusion of her autistic adult child in the decision-making process.

“*…[The PCP and staff are] trying to respect me, they're trying to respect him, I'm trying to respect him. So it's like a dance…he [has a conservatorship], but I don't really want to take him out of the conversation…that's difficult, to know what's really going on when everybody's trying to respect the rights…they want to ask him what he wants to do, which is lovely. But he's not capable of making that decision…it's nice that they ask, but sometimes, I wonder if they're actually taking that into consideration…I feel like it's great that they ask and it's interesting to see what he'll say, but what he'll say is, “I don't want to do the test.” But he needs a test…it also would be completely disrespectful if they didn't ask him.”*

##### Barriers for caregivers

Caregivers who accompanied their autistic adult child to primary care appointments were often frustrated and upset with policies that prohibited their full participation in the encounters. Some reported being denied health-related information or entry into the examination room because if the office does not “…have that documentation [guardianship forms] on file, they won't give you information. They'll look at date of birth, and they say, ‘They're over 18. We can't talk to you.”' Another caregiver explained that since her daughter turned 19, “…they don't let me into the back [and] that's when I get frustrated because I don't know what's going on…she just comes out and says, ‘Oh, that's it.' I'm like, what just happened?” Conversely, some caregivers lauded office policies and PCPs that included them in the appointments. One caregiver said that “no one has ever asked me to leave. I guess because it's kind of obvious that [my son] has autism. So maybe they want me there”, while another explained that if she did not have guardianship papers with her, she would feel comfortable telling the PCP, “Look–he is on the autistic spectrum, and I need to be there.”

#### Facilitators

One of the most commonly praised PCP attributes centered on the inclusion of autistic adults during primary healthcare interactions. Caregivers voiced that “if it's something [PCPs are] doing right that I've seen modeled it's…including him in the conversation…not being scared to communicate.” One mother said she appreciated when her PCP

“*…tries to talk directly to [my son]…in all the conversation[s]. Of course when it gets to the point where it's a little hard for him to answer or understand, or the doctor doesn't know…he comes to me. But, he tries to focus with [my son] first. And, I like that because he gives him the opportunity to express himself.”*

A PCP's ability to incorporate caregivers into their autistic adult child's care when appropriate was widely regarded as a facilitator to care. Caregivers ascribed the PCP “[spending] time with me and [letting] me be involved in all of [my son's] care” as a fundamental component of a successful primary healthcare encounter. As some caregivers reported experiences where PCPs expressed discomfort with their presence in the examination room, one caregiver specifically commended a PCP who was “very in tuned with me…I sat in on their initial doctor visits and held nothing back and he was comfortable, whether I was in the room or not. It didn't matter.”

Caregivers overwhelmingly highlighted that the best outcomes occurred when there was a mutual understanding that the physician was the medical expert and not an expert on the experiences or nuances of the autistic adult. This involved the PCPs willingness to listen to parents' advice on how best to work with their child and being “open to what I'm already doing and having successful results with.” One mother stated this entailed the PCP to “not be the expert anymore, and not be the tyrant ‘That this is the way it is'. But if they're willing to [listen to] your experiences, that would help.”

Some caregivers assumed a predominantly hands-off approach to promote their adult child's independence, acknowledging that they are of legal age to make their own health-related decisions. One caregiver mentioned they no longer accompany their son in the exam room “because he's 23 and I felt it would be inappropriate” while another said her daughter has the “final word” now that she is 18. Likewise, one caregiver who discussed her son's transition into adult life explained that

“*I will be there to support [my son] when I see he needs my help. All while helping him learn to make his own decisions. I don't want to decide in his stead…I want him deciding on his own like any other person.”*

##### Caregiver suggestions

Caregivers consistently reiterated that “adults with autism are there, and we must make them feel included in every sense of the word.” Caregivers said inclusive PCPs would greet their adult child by “simply saying to the patient, ‘Hello, how are you feeling today?' even if the individual is non-verbal” and throughout the exam would “be patient when they ask things and know that the autistic adult won't answer them or doesn't want to answer.” Caregivers wanted PCPs “to be a little bit more [understanding] and compassionate toward [the autistic adult] instead of just shutting them up”, thereby creating a space where their adult child “feels they can say or ask anything.” Caregivers recommended accomplishing this through “[telling] him what they're going to do and why”, “…[putting] medical terms…in a way that he can understand”, and “really respecting them. Listening…never [making] him feel rushed.” One caregiver described that an ideal PCP would be

“*Somebody who would take the time to really get to understand him. So, somebody who's going to go a little bit above and beyond and understand that this is somebody who can't process and kind of manage their own healthcare. They kind of need somebody to lead them a little bit. So, you know, having a doctor that I feel like I could send him to on his own, who's going to know how to handle him and give him the information that he needs.”*

### Stigma and assumptions about autism

#### Barriers

Many caregivers discussed the challenges associated with providers' incorrect assumptions about patient strengths and challenges as well as the stigmatizing reactions of staff members and other patients about autism. Some caregivers sensed that providers and staff were unaware of their autistic adult child's diagnosis because “he [physically] appears pretty typical” and “he gives the appearance of functioning in kind of a higher level than he does.” Caregivers also reported that their autistic adult child's physical appearance led people to discredit their disability, attributing behaviors to just being shy or outbursts to being under the influence of drugs and alcohol. One caregiver explained that “the challenge with these adults with autism [is that] they might look fine, they might be able to communicate, you know, for the most part [but] there's gaps.” This was considered a barrier to care when accommodations or extra help were not offered. As a result, a few caregivers said they always felt the need to explicitly explain to staff that their adult child had an autism diagnosis.

When challenging behaviors arose during primary healthcare encounters, caregivers described the “dirty looks” of healthcare staff, other patients in the office, and feelings that “nobody wants to sit next to you…it just doesn't feel great.” They also noticed that others were less tolerant of autistic adults regarding challenging behaviors, noting that “…it's a little more hard with an adult than with a little kid on the spectrum.” One caregiver reported that as a result of meltdowns,

“*We've had a terrible past with finding and keeping practitioners, because a lot of doctors have been nervous about my son's behavior…people are apparently so uncomfortable with his noises and his funny ways, that …they're not comfortable having him in their office…I can't tell you how upset it made me to have medical practitioners turn him away.”*

In contrast, one caregiver explained that the PCP's awareness of an autism diagnosis was challenging for her son, describing that he preferred when staff and providers were not aware he had autism. She described that

“*He understands and owns who he is, but I think it is embarrassing to him…he doesn't want people to label him disabled. You know, he has challenges, but he doesn't want people first off when he walks in the door [to say], “Oh, here's that high functioning autistic kid.” …And I think because…so much of his life has revolved around his disability, to him, it's not always the greatest experience to go to the doctor…[because] it's like not only is he not feeling well, but we've got to discuss his autism.”*

#### Facilitators

##### Caregiver suggestions

Caregivers reported no facilitators to care to combat the harmful effects of stigma and assumptions about autism during primary care encounters. However, they did offer suggestions for PCPs they believe might facilitate care in the future. Caregivers emphasized that PCPs should be understanding and accommodating of differences and disability—meaning being “aware of what our kids can and can't do”—without treating autistic adults like they are inferior to others. One caregiver explained that one of the most important things the PCP can do is make her son “feel like he's valued and that he's not being talked down to.” Additionally, caregivers described that their autistic adult child could sense when healthcare professionals had positive attitudes toward them and explained that “if they sense that the person is comfortable with them, it's going to make it that much easier.”

Ultimately, caregivers coveted PCPs who were accepting of “the positive and the negative” of autism. Caregivers expressed that “the spectrum is a special place and [a] specially ignored place” and communicated that acceptance from a PCP can simply look like an acknowledgment that “they are there, and they are just like the rest of us.”

### Caregiver experience

#### Barriers

Previous experiences of caregivers also had the potential to pose a significant barrier in the utilization of primary healthcare services for their autistic adult child. When reflecting on primary healthcare encounters, caregivers' experiences ranged from extremely stressful to, at minimum, “a chore we have to do”, with most caregivers reporting feeling “panick[ed]”, “alone”, and “scared” throughout the process. One caregiver described that many parents of autistic adults “suffer from stress, depression, anxiety.” Navigating the innately complex healthcare system was further complicated by topics requiring a high degree of health literacy, such as coordination of care and health insurance. Many caregivers reported a lack of confidence in their abilities to *coordinate their autistic adult child's care*, as evidenced by remarks such as “I'm the… case manager. And not a very good one all the time,” and “I wonder…if I somehow hamper the process…I don't know if I do.” When asked if having a professional case manager would ease the burden caregivers felt, one mother said, “…of course that will be a big help. We're talking about children here with a permanent disability. It's not just something that goes away after age 18.” Many caregivers echoed this sentiment and coveted assistance in care coordination and understanding the different roles of the healthcare team.

Several caregivers highlighted the importance of exhausting all resources for their autistic adult child; however, they often felt they had to laboriously advocate for *PCP referrals to specialists or necessary ancillary therapies*. Many caregivers explained that it felt like their concerns were not taken seriously, and PCPs often dismissed their request for testing, stating that “you don't need to put them through that. You don't need to do that,” even though the results were necessary in order to obtain referrals for needed resources. Other caregivers shared that “…even if I bring them a big, old packet of papers that she has all these symptoms and all these things going, and letters and referrals, they don't feel like she needs any services of any kind” if they don't present with the reported symptoms at the time of the appointment. Ultimately, caregivers explained that it seemed like their requests were frequently disregarded as PCPs stated the referral service was unnecessary, which prompted feelings of frustration and mistrust because “we're not going to take that they don't need to do it [new service/therapy] when we haven't heard from the doctor that actually specializes in this that there's no area of concern.” Conversely, a few caregivers reported that their PCPs were “…willing to give referrals anywhere…But I have to do the looking for the top doc in that area,” which created additional stress and responsibilities for the caregiver. Interestingly, caregivers explained that “when [your child is] young, it's so much easier…they [PCPs] understand your advocacy as a parent. It's pretty easy to get the team on board - it seems like it's just a little different [now that he's an adult].”

Several caregivers managing their autistic adult child's health reported dissatisfaction with *insurance*, specifically regarding the procedures related to obtaining coverage and access to desired physicians, as not having access to desired doctors was reported as extremely distressing to caregivers. They explained the need to rely on advice from other caregivers whose autistic adult child “had the same experience or procedure” in order to navigate the system. Researching different insurance plans and “learn[ing] a whole new system was anxiety-producing, with caregivers also reporting that “…there's too many choices. But you don't know which one is really relevant to you.” Caregivers described their personal challenges with insurance while simultaneously considering what the experience would be like for their autistic adult child–“I just think to myself, if [my son] had to do this himself, good Lord.”

Caregivers also reported feelings of stress trying to manage their autistic adult child's *challenging behaviors*, which was amplified by a lack of PCP-initiated strategies and assistance. One caregiver explained “…those providers that rely too much on me to [to provide strategies and] prepare [my son] for what they need to do…just adds to my own stress” while another said she has to “…help them [the PCP] to do the job, because they freak out, like, oh my God, I don't know how to treat this kid.” Another mother stated that “I have not, in a non-Autism related practice, ever seen anyone try any strategy whatsoever. In fact, they wouldn't even know a strategy if I told them what to do.” Interestingly, one mother described that the PCP provided accommodations for her son when he was younger, but that they were no longer offered to him as an adult. Caregivers reported feeling overwhelmed by staff not knowing how to interact with autistic adults. This was exemplified by a mother who explained that

“*Sometimes they're mean and they're not understanding that, like I said, she is very sensitive. “Didn't I tell you to sign right here?” There's just - she doesn't understand that, okay, you only told her once. She probably didn't hear it because she was spacing out. Sometimes she cries. And sometimes she just gets really angry and she just stays quiet. It hurts. And it makes me angry too… Especially when she takes it out on me. So, it's hard. Like, I wish I can just take it all away, but I can't.”*

Conversely, one caregiver described “I think I'd prefer to deal with it myself because, I mean, even if somebody is familiar with kids on the spectrum - you know, it's not like one strategy works for every kid. So, generally I think - I personally feel that it's better if the parent just … handles it themselves.”

### Facilitators

Caregivers did not report any specific facilitators for any of the barriers reported above. However, a few caregivers did comment on the importance of sharing resources and information with other caregivers in order to reduce the burden of navigating the healthcare system, so “other parents [won't] go through what we went through; all the roadblocks, all the hurdles and everything.” One caregiver described her motivation for providing support to other parents of autistic adults, stating:

“*I remember how it felt when no one helped me…I always get emotional when I think about that...You feel alone. There's nothing; there's no answers. No one to help you. Nobody knows what you're talking about...When I see other parents - I mean, now that he's older, he's an adult, and our younger kids [with autism] reap all the benefits of everything he went through - it's easier.”*

#### Caregiver suggestions

Caregivers described the desire for families to be trained to face barriers in the healthcare system, with one mother describing that “I'd like it if at school they gave lessons or if there were meetings about education for parents, due to the fact that parents need education to cope with a kid's situation.” Caregivers also reported that care coordination could be improved by simplifying the pre-visit process and receiving extra support from staff. Appointment-making logistics were described as complicated and inaccessible, both for their autistic adult child and themselves. They voiced that scheduling appointments online or *via* text would make it easier for their autistic adult child to be independent and make their own appointments; additionally, it would also be beneficial to caregivers with social anxiety and/or within the broad autism phenotype. For example, one mother revealed that

“*I've been observing this population for 20 years now, and I would say that knowing that there is a genetic link through parents, it's been my observation that many of our autistic friends' parents have some issues themselves. And social anxieties and such like that. I - and I know that I prefer to do things by text or online rather than talk on the phone.”*

Many caregivers desired a PCP who knew when to *refer the patient to a specialist* and could recognize signs that outside services might benefit their autistic adult child. They felt a PCP should have the insight to accept when issues were beyond their scope of practice and the wherewithal to guide patients toward the best treatment and specialty care. One caregiver explained that

“*I would say they'd have to be pretty much an out-of-the-box thinker and I don't think that they all have to be an expertise [sic] and everything, but they have to have a good scope that, 'This patient needs a little more than what I'm able to give' and pass that patient on.”*

Lastly, caregivers desired extra support, specifically in order to safely restrain their adult child if necessary during medical procedures. One caregiver explained that her husband “used to be able to do a lot of things with his daughter…But with time, she's grown up now. She's taller than me. It's very difficult.” Another parent had a similar experience where

“*[My son] had to have - last year, and the previous year, and the previous year before that, [he] had to have at least five other nurses on him, including [his father] to hold his arm and get a blood test. Not to kick, not to move, not to - you know, he's [more than] 200 pounds - he's 5'8* ½*.”*

### The impact of culture and ethnicity on care

#### Barriers

Over half of the caregivers who described their ethnicity as Hispanic/Latino reported feeling that PCPs looked at and/or treated them differently because of their ethnicity. A few caregivers perceived that this differential treatment was the result of PCPs incorrectly assuming they would have difficulty communicating in English, while several other caregivers placed the blame on themselves, noting that it was “partly also because we say nothing.” These caregivers explained they were hesitant to voice their opinions during primary healthcare encounters because they felt intimidated by English and American culture, were self-conscious to speak “broken English,” and were fearful of disrespecting the doctor. One caregiver explained that

“*What we Latin Americans almost always have is that we stay silent. We never ask because of shame, because of language…Sometimes we're more worried to think that they will give us a hard look, because the doctor often tells you something and you don't understand and sometimes you don't ask back out of shame. How will you show him that you didn't understand or that you're [not] dumb? And instead of showing that, you'd better not ask…So, we're more concerned about not being seen as dumb than about asking…And doctors always hold on to that - “this one's not even asking” - and they do things quickly with you. I mean, they don't put the same interest. And they don't because we allow it.”*

Language barriers between Spanish-speaking caregivers and PCPs existed even when interpreters were available and utilized. Caregivers reported challenges with the quality of the translation, the limited amount of time interpreters spent with them, and the lack of interpreters. Caregivers preferred to speak directly to a doctor fluent in Spanish rather than use an interpreter due to concerns with the quality and nuance of the translation “because in health you may understand one thing and it's the other. And that's actually a very delicate thing that has to be said properly.” Another caregiver pointed out that emotions get lost in translation when using an interpreter to communicate with the doctor, explaining that

“*You lost the feeling, you know, when you connect to the doctor [through an interpreter] with who's [sic] important. The messages don't go straight. When you talk, they go straight. And he feels your worry, your passion, whatever it is, you know? But when you translate, when he get there he's distracted because he had to wait while I finish to talk, and later she can understand a little bit what I said and translate only what she understand or what she hearing. And the whole message is lost, you know?”*

One caregiver recounted how she was initially denied an interpreter by a doctor who said “You don't need it, I understand you,” necessitating an argument to convince the PCP that he did not. As a result of language barriers, caregivers felt it was important for them to learn or improve their English to best support, care for, and navigate the health system on behalf of their autistic adult child.

#### Facilitators

Hispanic/Latino caregivers reported that the most salient facilitator to high-quality and culturally responsive care was when a PCP spoke Spanish. Some caregivers even noted that they appreciated when their non-fluent PCP attempted to communicate with them in Spanish, perceiving it as respectful and describing it as a facilitator to an improved experience although not necessarily leading to improved care. Despite highlighting numerous challenges with interpreters, caregivers were grateful for offices that employed bilingual staff members and PCPs who initiated recruiting interpreters into appointments. Some caregivers believed that challenges with language barriers could be reduced if PCPs dedicated longer amounts of time speaking with and clarifying information to patients and caregivers in both English and Spanish. For example, one caregiver described that:

“*…[The PCP is] speaking English with [my son]…I speak English a little bit, but no real. But when it's very important, I ask the doctor in Spanish. And [he] explain to me in Spanish when I don't understand, you know? …I confirm in Spanish. And he have the time to do that for me, too***… ***I feel so lucky, you know? To have this kind of doctors.”*

## Discussion

Overall, findings from this study suggest that autistic adults face a multitude of barriers related to primary care health encounters and that the receipt of high-quality care is hindered by a dearth of strategies or interventions to improve that care. Previous research has described three interwoven factors–the patient, the PCP, and the overall healthcare system–that have both individual and cumulative potential to affect the experiences of autistic consumers during healthcare encounters (11, 36). Findings from the current study support the existence of and the complex interplay between these three factors within our seven themes.

For example, caregivers repeatedly emphasized the importance *of minimizing waiting* as they perceived this to be anxiety-inducing and “the worst part of the whole experience” for their autistic adult child. Although similar complaints by autistic adults and caregivers have been previously reported (15, 16, 36), solutions to address this barrier are limited. They include the use of sensory-based accommodations (e.g., dimming the waiting room lights) and bypassing the waiting room altogether by remaining in the car or outside the office until the doctor was ready to see them (15, 26, 37). In addition to these previously reported strategies, participants in this study attempted to navigate this challenge by strategically scheduling the first or last appointment of the day. PCPs have also recognized that long wait times are problematic, noting system-level challenges which prohibit accommodations, including monitoring the amount of time spent waiting in the waiting room and scheduling sufficient “blocks of time” for the visit (13).

Consistent with previous research, caregiver participants in this study emphasized that *incompatible communication styles* between patient and provider were problematic in a myriad of ways. For example, autistic adults have reported that providers did not use accessible language, spoke quicker than autistic adults could process, and were unwilling to accommodate different forms of communication (i.e., writing) (11, 12, 36). A supporter of an autistic adult in one study explained that it was valuable when a provider inquired with the patient about their communication preferences *via* email prior to the visit (11). In addition, participants felt the limited amount of time spent with providers did not allow the opportunity for all questions to be addressed, thereby contributing to communication challenges (12). PCPs recognize the benefits of allotting extra time with autistic patients, but cited financial disincentives as a strong deterrent (13), a barrier also noted by providers in pediatric settings (38, 39).

Barriers in the *sensory environment* of the primary care setting were reported in this and several other studies (11, 12, 15, 16); however, minimal action has been taken to address this widespread concern. Caregivers participating in this study suggested that modifications to the sensory environment, such as creating a separate “quiet room” to wait in or providing weighted blankets, could help ease the anxiety of their autistic adult children. Certain disciplines, such as occupational therapy, have a long and rich history of examining the modification of characteristics of the environment in order to improve experiences for autistic individuals as well as those with other sensory processing difficulties (40). For example, adapting the sensory environment of the dental office was found to minimize behavioral and physiological distress during care for children and adults with autism and intellectual and developmental disabilities (41–43). As the built environment of healthcare settings has the potential to influence service delivery and individual health outcomes (44, 45), it is imperative that considerations take into account the more varied sensory aspects of the environment, beyond the traditional acoustics, temperature, and visual properties (e.g., artwork) which may be particularly salient to autistic patients.

PCPs report feeling apprehensive about which specialists to refer to when they have questions about the developmental needs of autistic individuals (23) as well as a lack of knowledge about autism-specific referral pathways (25), an inevitable barrier considering that services for autistic adults are not adequately publicized (14). The inclusion of interdisciplinary professionals on the primary healthcare team–as suggested by the caregivers in this study–may pose a solution to this barrier. Team members with knowledge about autism-specific services may assist PCPs with care coordination and referrals to outside services (46). Programs such as ECHO, a 12-week informational session connecting PCPs to an interdisciplinary expert team, has shown promise in improving PCPs' understanding of resources for autistic adults and increasing local access to community resources (27).

Caregivers in this study described that autism-related stigma from healthcare professionals and others in the office served as a barrier to care. It is not surprising that a high proportion of PCPs are unaware they treat autistic patients (14), as autistic adults have reported hesitancy to disclose their diagnosis to providers out of fear of receiving inferior care than their non-autistic counterparts (11, 12, 47). In addition, one caregiver in this study explained how she believed her son's internalized stigma led him to not report his diagnosis to his PCP. Additionally, many PCPs report a lack of understanding and training regarding treating autistic patients (13, 14, 25). Limited knowledge about autism is correlated with increased stigma (48), which can threaten health outcomes for these patients and strain patient-provider relationships. Continuing medical education for providers can increase knowledge about autism and act as a facilitator to care (13, 14); in fact, a small number of medical schools have recently incorporated training specific to treating adults with intellectual and developmental disabilities, including autism, into their curriculum through preclinical and clinical elective courses (49).

Multiple surveys also suggest that PCPs commonly feel ill-equipped to provide the necessary emotional and behavioral supports for their patients and implement appropriate accommodations (23, 25, 50). Caregivers in this study suggested that incorporating interdisciplinary healthcare team members into primary care examinations may support PCPs in providing individualized, holistic care by addressing the social-emotional and sensory needs of autistic adults. Due to the inadequacies of the current system to meet the needs of autistic adults, caregivers proposed a “facilitated facility” knowledgeable, interdisciplinary, and experienced in the identification and implementation of accommodations as an alternative form of service delivery. A recent study found that a patient-centered medical home has the potential to meet the complex physical and mental healthcare needs of autistic adults by providing comprehensive, coordinated, and accessible patient-centered care (51). A promising example of the medical home model of care is the Achievable Foundation^3^, a federally qualified health center in California that offers medical services uniquely suited to meet the needs of children and adults with intellectual and developmental disabilities. The Achievable Foundation aims to minimize barriers in primary care at the patient, provider, and system level by employing PCPs with expertise caring for this population, an accessible facility to reduce patient anxiety, longer appointment lengths, and on-site specialty services such as mental health and neurology. Another alternative model of service delivery that may address these barriers is telehealth. Interviews with autistic adults and caregivers of autistic adults found that primary care delivered *via* telehealth increased patient comfort by eliminating exposure to the overstimulating clinical environment and afforded comparable or improved patient-provider communication (52). The CAST (Center for Autism Services and Transition) clinic, which was built into a larger primary care practice, uses patient-centered medical home principles and offers telehealth to improve care for autistic adults (53). Billing and reimbursement for hallmarks of the program such as extended appointments and “happy visits” have been a challenge, requiring the program to rely heavily on grant and donor funding (53). When these alternative forms of service delivery are not available, financially feasible, or appropriate, providing PCPs with individualized accommodation reports have been reported by patients and providers to reduce barriers (26).

Latino patients as well as experts on health disparities report that Latinos receive inferior care to non-Latino white patients (54, 55). Over half of the caregivers who described their ethnicity as Hispanic/Latino in this study reported feeling that they received lower quality care compared to their non-Hispanic/Latino counterparts, due to communication barriers and PCPs failing to provide culturally responsive care. Caregiver participants reported that quality of care might also be diminished “partly also because we say nothing,” which is consistent with previous literature suggesting that the experience of common Latino cultural tenets of *respeto* and *confianza* can influence families to be “grateful and not complain,” even if they are seemingly subjected to inadequate care (56). According to the 2020 census, Hispanic/Latino individuals account for 18.7% of the United States population (57), so it is essential to reduce the immense health disparities faced by Hispanic/Latino autistic adults whose health inequities are shaped by the cumulative effects of belonging to two marginalized groups.

Additionally, some studies have suggested that the interplay between barriers can further intensify them. For example, autistic adults reported that the distress they experienced from sensory overstimulation in the clinical environment made it even more difficult to communicate with providers (12, 15), implying that environmental modifications may indirectly facilitate communication. This is also supported by the interwoven nature of the themes reported in this study. For example, the lack of provider expertise to support autistic patients' management of anxiety during visits–a common complaint by caregivers in this study–intensified barriers such as patient-provider communication, sensory-related discomfort due to the clinical environment, and also negatively impacted caregiver experience. Conversely, this study also found that strategies aimed to facilitate one challenge could simultaneously also improve other barriers unintentionally. For example, caregivers reported that when PCPs implemented communication techniques such as narrating the physical examination, it provided other downstream improvements such as overall patient-provider communication as well as patient anxiety.

Despite the fact that the average lifespan lasts longer in adulthood than in childhood, the attention of autism-related research dedicated to adults compared to children is strikingly disproportionate (58). Consequently, supports and services available for autistic adults—including primary care-related interventions—are significantly rarer and less accessible than for children with autism (13, 58). Research focusing on adults is essential because autism can present differently across the life span, necessitating distinct interventions for different stages of life (58).

### Strengths and limitations

The data presented here is part of a larger study that included qualitative interviews with autistic adults, caregivers, and PCPs. The majority of caregivers in this study were recruited when their autistic adult child could not participate in an interview verbally, written, or utilizing augmentative and alternative communication (AAC) strategies, in order to obtain information about primacy care health experiences of autistic adults–albeit from the perspective of the caregiver. Four autistic adults requested their caregiver also be interviewed and an additional four autistic adults were unable to respond to interview questions after consenting to participate; to honor these requests and collect information from all families, there are a small number of dyads present in the overall data (only caregiver data is reported here). Therefore, this sample represents the experiences of caregivers whose autistic adult child required their assistance to engage in healthcare encounters, and the conclusions formed are centered on caregiver experience, perspectives, and opinions and may not be indicative of the experiences of autistic adults themselves. The experiences of autistic individuals who do not require or want the help of a caregiver may be more accurately reflected in the analysis of the interviews with autistic adults, and future work should combine all participant voices–autistic adults, caregivers, and PCPs–in order to develop a comprehensive perspective of barriers and facilitators to care.

## Conclusion

Overall, this study identifies barriers to the utilization and receipt of primary care for autistic adults and offers tangible solutions, from the perspective of caregivers, to improve care. Given the heterogeneous nature of autism, the inclusion of caregivers' voices is imperative to fully encompassing and understanding primary healthcare encounters for autistic adults who are not otherwise able to or choose not to report for themselves. The results of this study are largely congruent with previous research reporting on primary care health encounters from autistic adults and PCP perspectives. Ultimately, there are many barriers to high-quality care and few facilitators to care; however, caregivers in this study proposed valuable suggestions to improve primary care experiences that should be considered when informing future service delivery and intervention development.

## Data availability statement

The dataset presented in this article are not readily available because our study analyzed qualitative data which contain potentially identifiable information and participants did not consent to having their full transcripts made publicly available. Due to ethical restrictions imposed by our IRB surrounding sensitive data and participant privacy concerns, we are unable to make the transcripts available publicly. Excerpts of the transcripts relevant to the study are available to anyone who contacts us with this request. Requests to access the dataset should be directed to lstein@chan.usc.edu.

## Ethics statement

The studies involving human participants were reviewed and approved by Institutional Review Board of the University of Southern California Health Sciences (HS-17-00477). The patients/participants provided their written informed consent to participate in this study.

## Author contributions

All authors made a significant contribution to this work in multiple aspects of the development of this study and manuscript, including the conception, study design, execution, acquisition of data, analysis and interpretation, drafting and critical review of the article, have provided final approval of the manuscript, and agree to be accountable for the content of the work.

## Funding

This study was supported by the American Occupational Therapy Foundation (AOTF IRG16DUKER) and the National Institute of Child Health and Human Development (K12 HD055929).

## Conflict of interest

The authors declare that the research was conducted in the absence of any commercial or financial relationships that could be construed as a potential conflict of interest.

## Publisher's note

All claims expressed in this article are solely those of the authors and do not necessarily represent those of their affiliated organizations, or those of the publisher, the editors and the reviewers. Any product that may be evaluated in this article, or claim that may be made by its manufacturer, is not guaranteed or endorsed by the publisher.

## Footnotes

^1^We would like to recognize that current explorations of the language preferences of the autistic community reveal discrepancies between use of identify-first language and person-first language. Many autistic individuals and self-advocates prefer identify-first language (i.e., autistic individual), whereas healthcare professionals and parents are more likely to utilize person-first language (i.e., individual with autism) (see text footnote 2). For dissemination of the information in this manuscript, we chose to use identify-first language to honor the expressed preferences of many in the autism community, including autistic scholars, researchers, and advocates (see text footnote 2). The majority of caregivers in this study referred to their children using person-first language (i.e., “my son with autism”). We have chosen to keep the quotes of the caregivers intact as to honor the authentic voices and respect the language choices of our participants.

^2^ https://www.liebertpub.com/doi/10.1089/aut.2020.0014

^3^ https://achievable.org/patient-care/
